# Patient-centered integration of the treatment and prevention of cardiovascular diseases in the community: a digital intelligence exploration

**DOI:** 10.3389/fmed.2025.1593238

**Published:** 2025-07-08

**Authors:** Zhaoyuan Zhou, Xiaotong Gao, Meichen Jiang, Jiachun Wang, Qiang Zhang, Zhen Wang, Zhe Yan, Kai Yu, Xinyue Chen, Xiaoqing He, Shuang Wang

**Affiliations:** Department of General Practice, The First Affiliated Hospital of China Medical University, Shenyang, Liaoning, China

**Keywords:** primary healthcare, general practitioner, elderly stroke, patient-centered treatment, digital platform

## Abstract

**Background:**

We aimed to explore the benefits of the “Digital and Intelligent Patient-centered Integration of Treatment and Prevention for Cardiovascular Diseases in the Community” (DITP), which is a new model for managing cardiovascular disease risk factors in high-risk elderly stroke patients.

**Methods:**

A total of 338 elderly high-risk stroke patients were included and subjected to a 6-month self-controlled DITP study.

**Results:**

The five chronic diseases with the highest prevalence rates were hypertension (89.94%), dyslipidemia (73.37%), overweight (65.70%), diabetes (52.96%), and atrial fibrillation/valvular heart disease (2.07%). Moreover, 86.7% of patients had two or more simultaneous chronic diseases, and 27.22% had a history of smoking. After 6 months of intervention, (1) with respect to the control of multiple risk factors, there were improvements in blood pressure and blood lipid compliance rates. The blood glucose compliance rate (50.30% preintervention vs. 62.43% postintervention) and the optimal risk factor control rate (3.85% preintervention vs. 8.88% postintervention) were significantly improved (P < 0.05). (2) Regarding physiological indicators, fasting blood glucose (7.44 ± 2.37 mmol/L preintervention vs. 6.99 ± 2.15 mmol/L postintervention), cholesterol (5.56 ± 1.3 mmol/L preintervention vs. 5.20 ± 1.22 mmol/L postintervention), and low-density lipoprotein cholesterol levels significantly decreased (*P* < 0.05). (3) The proportions of patients with good medication adherence scores for hypoglycemic (6.76 ± 1.76 preintervention vs. 7.19 ± 1.24 postintervention), lipid-lowering (6.80% preintervention vs. 15.09% postintervention), and antiplatelet (4.73% preintervention vs. 7.69% postintervention) drugs significantly increased (*P* < 0.05). (4) The activation score and the proportion of the highest activation level significantly improved (*P* < 0.05). (5) Regarding major adverse cardiovascular events, 2 (0.59%) and 13 (3.85%) patients experienced myocardial infarction and cerebral vascular ischemia events, respectively. (6) Univariate analysis revealed that employment status, per capita monthly family income, hypertension status, diabetes status, family history of stroke, and hypoglycemic and lipid-lowering treatment had significant effects on the optimal risk factor control rate (*P* < 0.05). Multivariate logistic regression analysis revealed that employment status, nondiabetes status, a family history of stroke, and receiving lipid-lowering treatment were associated with better control.

**Conclusion:**

This DITP model will help actively improve the health of elderly high-risk stroke patients in the long term and should be further applied and promoted in primary healthcare in the future.

## 1 Introduction

Stroke is currently the leading cause of death among Chinese residents, with its prevalence remaining at a peak level (1). The number of first-time stroke cases and deaths from stroke remains high every year. In 2020, the estimated overall prevalence, incidence rate and mortality of stroke in mainland China were 2.6%, 505.2/100,000 person-years and 343.4/100,000 person-years, respectively (1–4). The most effective way to reduce the incidence of stroke is to strengthen primary prevention (5). In the past 50 years, studies on the prevention of stroke and atherosclerotic cardiovascular disease in Europe and the United States have shown that standardized drug treatment for hypertension, diabetes, and dyslipidemia, as well as chronic disease management, lifestyle interventions, risk assessment and health education, are key primary prevention strategies that can significantly reduce the mortality rates of stroke and cardiovascular diseases. Moreover, another study recommended a health services approach involving patient-centered and team-based care (6).

In recent years, online diagnosis and treatment has gradually become a popular global trend characterized by high convenience and ease of use, overcoming time limitations and space constraints. Through internet medical platforms, patients can receive medical services such as disease diagnosis, treatment advice, and prescriptions while carrying out remote monitoring and online consultation for patients to facilitate follow-up visits and health management (7). The high-risk population for stroke includes individuals with three or more stroke risk factors, mainly hypertension, diabetes, dyslipidemia, atrial fibrillation or valvular heart disease, smoking, obesity, lack of physical activity, and a family history of stroke (8). Online diagnosis and treatment can provide strong support and guidance for the primary prevention of this population, reducing patients’ medical costs and enabling broader access to high-quality medical resources (9–12). Patient-centered care, team-based care and internet medicine are suitable for primary care medical institutions with relatively scarce medical resources.

At present, for people at high risk of stroke, controlling hypertension rates and fasting blood glucose (FBG) levels in individuals with type 2 diabetes is the focus of primary care management (1–4). However, the health status of patients with multiple stroke risk factors, such as hypertension, diabetes, dyslipidemia, and atrial fibrillation, is often ignored in comprehensive prevention and treatment at the primary care level (13–15). There is an urgent need for stroke prevention among Chinese community residents, and the management of primary prevention for stroke should be greatly strengthened and prioritized at the primary care level. This study is the first to propose the Digital and Intelligent Patient-Centered Integration of Treatment and Prevention for Cardiovascular Diseases in the Community (DITP) to explore its benefits for elderly high-risk stroke patients.

## 2 Materials and methods

### 2.1 Study design

For the present study, we retrospectively selected one community health service center from 14 cities in Liaoning Province from June 2023 to December 2023 and included a total of 338 research subjects. The community health service center selected by this research institute is representative of the local area and includes well-equipped medical equipment, professional family doctors familiar with the local residents’ situation, frequent interactions with higher-level hospitals, maintaining stable and close collaborative relationships, and possessing high-quality primary healthcare capabilities. The intervention included a 6-month follow-up using the DITP intervention and self-control methods. This study adhered to the principles of the Helsinki Declaration and was approved by the Institutional Review Board (AF-SOP-07-1.1-01).

### 2.2 Participants

The inclusion criteria were as follows: (1) signed contracts with primary care family doctors; (2) aged 65–79 years; (3) at high risk for stroke, as defined by the “National Health Commission Stroke Prevention and Treatment Engineering Committee Stroke High-Risk Population Screening and Intervention Project: those with three or more of the eight stroke risk factors, including hypertension, dyslipidemia, diabetes, atrial fibrillation or valvular heart disease; smoking history; apparent overweight or obesity status; lack of physical activity; and family history of stroke; and (4) provided informed consent.

The exclusion criteria were as follows: (1) did not meet the inclusion criteria; (2) had concurrent participation in other similar projects; (3) did not have transient ischemic attacks or a history of stroke (because this project is a primary prevention study for stroke); and (4) were unable or refused to participate in this project due to illness or other reasons.

### 2.3 Data collection

#### 2.3.1 Questionnaire

(1) Self-made survey questionnaire: I. Personal basic information: name, sex, age, contact information, long-term residence, education level, marital status, employment status, per capita monthly income of the family, medical insurance reimbursement method, etc.; II. Family history of stroke; III. Lifestyle: smoking habits, physical exercise habits; IV. Main medical history and control conditions: cerebrovascular disease, heart disease, hypertension, dyslipidemia, abnormal glucose metabolism, etc. V. Medication situation.

(2) Medication Adherence Scale: The Morisky Medication Adherence Scale-8 (MMAS-8) (16) and its Chinese revision (17) were used for evaluation. The maximum score for the scale is 8 points. Poor compliance: < 6 points; moderate compliance: 6- < 8 points; good compliance: 8 points. It has good reliability and validity.

(3) Patient Activation Measure (PAM): The Chinese version (18) of the Patient Activation Measure (PAM) developed by the University of Oregon Hibbard et al. (19) was used for evaluation. The scale assigns scores to each item based on values of 0, 1, 2, 3, and 4, with a total of 22 items. The total score obtained from the scale is the original score, which is then converted into the patient’s activation score based on the original score. The range is 0–100 points. Higher scores indicate a higher level of patient activation. PAM scores can be categorized into four levels: Level 1: ≤ 47.0 points; Level 2: 47.1–55.1 points; Level 3: 55.2–67.0 points; and Level 4: > 67.1 points. It has good reliability and validity.

Prior to the investigation, all the researchers were trained to ensure the accuracy and completeness of the questionnaire.

Physical examination included height, weight, body mass index (BMI), and blood pressure. The laboratory tests included four items related to blood lipids (total cholesterol, TC; triglycerides, TG; low-density lipoprotein cholesterol, LDL-C; high-density lipoprotein cholesterol, HDL-C) and FBG.

### 2.4 Specific implementation plan

**FIGURE 1:**
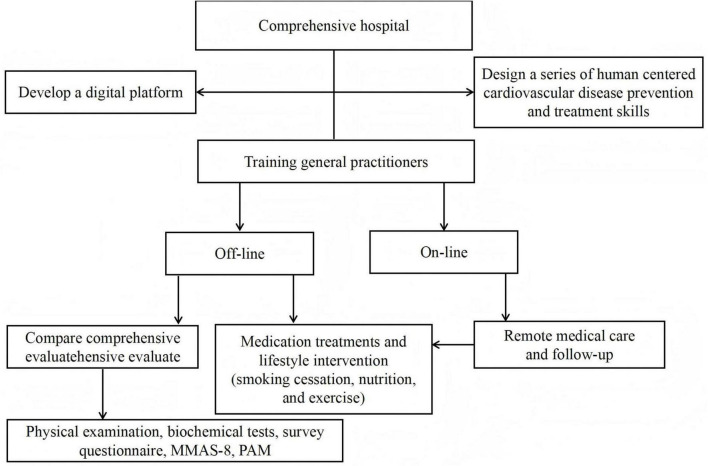
The digital empowerment of the patient-centered cardiovascular disease risk factor comanagement model.

The digital empowerment of the patient-centered cardiovascular disease risk factor comanagement model developed by our team for managing cardiovascular disease risk factors in high-risk stroke populations is as follows: (1) Our team designed a series of patient-centered cardiovascular disease prevention and treatment skills based on comprehensive hospitals and provided professional training for general practitioners in community health service centers. (2) We subsequently developed a digital, patient-centered online management platform for cardiovascular disease risk factors, wherein trained general practitioners can collect patient information and upload it to the platform for health data management. Specifically, DITP is an application, a mobile app. Health management can be carried out on this app, and patients’ physical examination reports and health assessment results can be uploaded. Moreover, general practitioners can develop personalized health management goals, treatment and follow-up plans for patients on the app and provide personalized health consultation, health education and regular follow-up through its chat function. (3) Offline, general practitioners conduct consultations with patients; comprehensively evaluate them through physical examinations, biochemical tests, survey questionnaires, medication adherence scales, and patient activation measurement scales; and provide lifestyle interventions and medication treatments. Online, general practitioners provide remote medical care and follow-up to patients through digital platforms, including guidance on nonpharmacological treatments such as smoking cessation, nutrition, and exercise, as well as urging and guiding patients on medication. General practitioners conduct weekly follow-up visits to patients. Through this combined online and offline management model, our team provides patients with individualized, patient-centered treatment (Figure 1).

### 2.5 Outcome statistics and evaluation

The main outcome indicator of this study is the optimal control rate for risk factors, which is indicated by simultaneously meeting the control standards for blood pressure, FBG, low-density lipoprotein cholesterol, and nonsmoking (20, 21). The detailed criteria were as follows: ① Blood pressure < 140/90 mmHg; ② fasting blood glucose < 7.00 mmol/L (126 mg/dL); ③ nonsmoking status; and ④ according to the risk of ASCVD, patients with different levels of blood lipid targets are classified as follows: low-risk patients with LDL-C < 3.4 mmol/L; LDL-C < 2.6 mmol/L in patients with medium- and high-risk or low- and medium-risk diabetes; LDL-C < 1.8 mmol/L in extremely high-risk patients.

The secondary outcome measures included the following: (1) evidence of stroke-modifiable risk factor compliance rates, including the blood pressure control rate, blood glucose control rate, blood lipid control rate, nonsmoking rate, BMI qualification rate, and appropriate physical activity rate; (2) medication adherence; (3) patient activation; and (4) incidence of major adverse cardiovascular events.

### 2.6 Statistical analysis

Statistical analysis was conducted via SPSS 27.0 software to establish a research database and conduct statistical analysis. Categorical variables are represented by the frequency (n) and composition ratio (%), whereas numerical variables are represented by the mean ± standard deviation or interquartile range (IQR). Paired-samples t tests were used for comparisons of means ± standard deviations, and paired-samples rank-sum tests were used for data that did not meet the criteria of independence, normality, and homogeneity of variance. Chi-square tests were used for comparisons of frequencies and percentages. Univariate analysis was performed on the factors that affect the optimal control of stroke risk factors. A multicollinearity test was performed on the significant influencing factors in the univariate analysis, and then independent variables with tolerance > 0.1 or variance inflation factor (VIF) < 15 were included in the multiple logistic regression analysis. P < 0.05 was considered statistically significant.

## 3 Results

### 3.1 Basic characteristics of the high-risk stroke population

#### 3.1.1 Basic information of the high-risk stroke population

This study included 338 individuals at high risk for stroke, and after 6 months of intervention, 338 valid questionnaires were collected, for an effective rate of 100%. The age range of the high-risk stroke population included in this study was 65–79 years, with an average age of 70.36 ± 3.92 years. There were 149 males (44.08%) and 189 females (55.92%). The specific social demographics of the patients are shown in Table 1.

**TABLE 1:** Demographic characteristics of the participants.

Characteristic	Category	Frequency	Percent (%)
Sex	Male	149	44.08
	Female	189	55.92
Long term residence	Rural area	7	2.07
	City	331	97.93
Age (years)	65–74	272	80.50
	75–79	66	19.50
Marital status	Married	319	94.38
	Widowed/divorced/ single	19	5.62
Education degree	Elementary school/below	28	8.26
	Middle school	239	70.70
	High school/vocational school	46	13.57
	College/bachelor’s degree/above	25	7.37
Employment status	Employed	61	18.05
	Retired	277	81.95
Per capita monthly household income (yuan)	≤2,000	32	9.47
	2,001–4,000	255	75.44
	4,001–8,000	49	14.50
	> 8,000	2	0.59
Medical insurance type	Urban resident medical insurance	41	12.13
	Urban employee medical insurance	267	78.99
	New rural cooperative medical insurance/Others	30	8.88

#### 3.1.2 Distribution characteristics of risk factors in the high-risk stroke population

In this study, the five chronic disease incidence rates from highest to lowest were hypertension (304 patients, 89.94%), dyslipidemia (248 patients, 73.37%), overweight (222 patients, 65.70%), diabetes (179 patients, 52.96%), and atrial fibrillation or valvular heart disease (7 patients, 2.07%). Among them, 293 patients (86.7%) had more than two chronic diseases, and 92 patients (27.22%) had a history of smoking. The specific distribution characteristics of the risk factors are shown in Table 2.

**TABLE 2:** Distribution characteristics of risk factors in the high-risk stroke population.

Characteristic	Category	Frequency	Percent (%)
Hypertension	Yes	304	89.94
	No	34	10.06
Diabetes	Yes	179	52.96
	No	159	47.04
Dyslipidemia	Yes	248	73.37
	No	90	26.63
Atrial fibrillation/valvular heart disease	Yes	7	2.07
	No	331	97.93
Family history of stroke	Yes	76	22.49
	No	262	77.51
Overweight	Yes	222	65.70
	No	116	34.30
Smoking status	Never smoked	246	72.78
	Currently smoking	59	17.46
	Quit smoking	33	9.76
Lack of physical activity	Yes	224	66.27
	No	114	33.73
Suffering from two or more chronic diseases	Yes	293	86.69
	No	45	13.31

### 3.2 Comparison of stroke risk factor control in the high-risk stroke population before and after intervention

**FIGURE 2:**
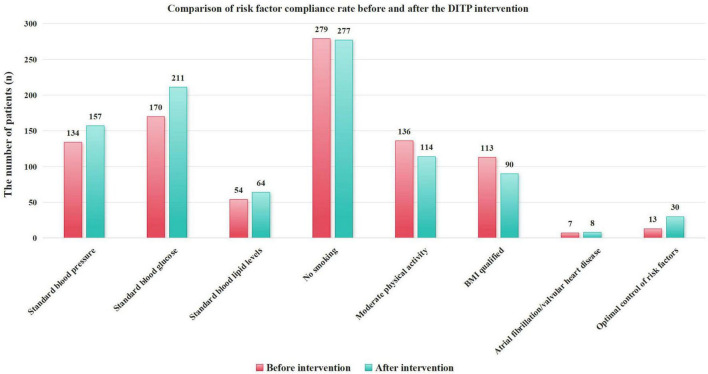
Comparison of risk factor compliance rates before and after the DITP intervention.

After 6 months of intervention, the blood pressure control rate increased from 39.64 to 46.45%, with an improvement rate of 6.80%. The blood glucose control rate increased from 50.30 to 62.43%, with an improvement rate of 12.13%. The blood lipid control rate increased from 15.98 to 18.93%, with an improvement rate of 2.96%. The optimal control rate for risk factors increased from 3.85 to 8.88%, with an improvement rate of 5.03%. The differences in the blood glucose control rate (χ^2^ = 10.110, P = 0.001) and optimal control rate for risk factors (χ^2^ = 7.177, P = 0.007) before and after the intervention were statistically significant. The detailed results are shown in Table 3 and Figure 2.

**TABLE 3:** Comparison of risk factor control compliance in high-risk stroke patients before and after the intervention.

Compliance rate of risk factors	Before intervention, n (%)	After intervention, n (%)	Improvement rate (%)	χ^2^	*P*-value
Standard blood pressure	134 (39.64)	157 (46.45)	6.80	3.192	0.074
Standard blood glucose	170 (50.30)	211 (62.43)	12.13	10.110	0.001*
Standard blood lipid levels	54 (15.98)	64 (18.93)	2.96	1.027	0.311
No smoking	279 (82.54)	277 (81.95)	−0.59	0.041	0.840
Moderate physical activity	136 (40.24)	114 (33.73)	−6.51	3.072	0.080
BMI qualified	113 (33.43)	90 (26.63)	−6.80	3.724	0.054
Atrial fibrillation/valvular heart disease	7 (2.07)	8 (2.37)	−0.30	2.853	0.415
Optimal control of risk factors	13 (3.85)	30 (8.88)	5.03	7.177	0.007*

BMI, body mass index; *P < 0.05.

### 3.3 Comparison of physiological indicators of the high-risk stroke population before and after intervention

After 6 months of intervention, the patients’ FBG (*t* = 3.167, *P* = 0.002), TC (*t* = 4.398, *P* < 0.001), TG and LDL-C decreased compared with those before intervention. The differences before and after the FBG and TC interventions were statistically significant. The detailed results are shown in Table 4.

**TABLE 4:** Comparison of physiological indicators before and after intervention.

Characteristic	Before intervention (*n* = 338)	After intervention (*n* = 338)	*T*-value/*z*-value	*P*-value
SBP	145.73 ± 18.43	149.40 ± 22.29	−2.841	0.005
DBP	82.86 ± 10.19	84.12 ± 10.84	−1.738	0.083
FBG	7.44 ± 2.37	6.99 ± 2.15	3.167	0.002*
TC	5.56 ± 1.3	5.20 ± 1.22	4.398	< 0.001*
TG (IQR)	1.71 (1.14, 2.51)	1.64 (1.14, 2.26)	1.682	0.092
HDL-C	1.46 ± 0.43	1.41 ± 0.4	1.469	0.143
LDL-C	3.31 ± 1.1	3.22 ± 1.07	1.206	0.228
BMI	25.34 ± 3.09	25.58 ± 3.16	−1.577	0.116

SBP, systolic blood pressure; DBP, diastolic blood pressure; FBG, fasting blood glucose; TC, total cholesterol; TG, triglyceride; HDL-C, high-density lipoprotein cholesterol; LDL-C, low-density lipoprotein cholesterol; BMI, body mass index; IQR, interquartile range; *P < 0.05.

### 3.4 Comparison of medication adherence among the high-risk stroke population before and after intervention

#### 3.4.1 Comparison of medication adherence scores among the high-risk stroke population before and after the intervention

The antihypertensive medication compliance score increased from 6.46 ± 1.97 points to 6.73 ± 1.65 points. The hypoglycemic drug compliance score increased from 6.76 ± 1.76 points to 7.19 ± 1.24 points. The lipid-lowering medication compliance score increased from 6.35 ± 1.93 points to 6.75 ± 1.79 points. The hypoglycemic medication compliance score significantly improved after the intervention (*t* = −2.115, *P* = 0.036), as shown in Table 5.

**TABLE 5:** Comparison of medication adherence scores before and after the intervention.

Medication types	Adherence score preintervention	Adherence score postintervention	*T*-value	*P*-value
Antihypertensive drugs	6.46 ± 1.97	6.73 ± 1.65	−1.620	0.107
Hypoglycemic drugs	6.76 ± 1.76	7.19 ± 1.24	−2.115	0.036*
Hypolipidemic drugs	6.35 ± 1.93	6.75 ± 1.79	−1.192	0.237
Antiplatelet drugs	6.78 ± 1.56	7.03 ± 1.63	0.105	0.917

*P< 0.05.

#### 3.4.2 Comparison of medication adherence levels among the high-risk stroke population before and after the intervention

After 6 months of intervention, the proportion of patients with good compliance with antihypertensive medication increased from 23.37 to 24.85%, with an improvement rate of 1.48%. The proportion of patients with good compliance with hypoglycemic drugs increased from 17.16 to 21.89%, with an improvement rate of 4.73%. The proportion of patients with good compliance with lipid-lowering medication increased from 6.80 to 15.09%, with an improvement rate of 8.28%. The proportion of patients with good adherence to antiplatelet medication increased from 4.73 to 7.69%, with an improvement rate of 2.96%. The proportion of patients with good compliance with lipid-lowering drugs before and after intervention (χ^2^ = 13.845, *P* < 0.05) and the proportion of patients with good compliance with antiplatelet drugs (χ^2^ = 8.222, *P* < 0.05) were significantly different. The specific levels of medication adherence for various medications are shown in Table 6.

**TABLE 6:** Comparison of medication adherence levels among patients before and after the intervention.

Medication types	The medication adherence level	Before intervention N (%)	After intervention N(%)	Improvement rate (%)	χ^2^	*P*-value
Antihypertensive drugs	No	122 (36.09)	117 (34.62)	−1.48	5.619	0.132
	Poor	54 (15.98)	36 (10.65)	−5.33		
	Moderate	83 (24.56)	101 (29.88)	5.33		
	Good	79 (23.37)	84 (24.85)	1.48		
Hypoglycemic drugs	No	217 (64.20)	204 (60.36)	−3.85	6.355	0.096
	Poor	24 (7.10)	13 (3.85)	−3.25		
	Moderate	39 (11.54)	47 (13.91)	2.37		
	Good	58 (17.16)	74 (21.89)	4.73		
Hypolipidemic drugs	No	250 (73.96)	237 (70.12)	−3.85	13.845	0.003*
	Poor	24 (7.10)	23 (6.80)	−0.30		
	Moderate	41 (12.13)	27 (7.99)	−4.14		
	Good	23 (6.80)	51 (15.09)	8.28		
Antiplatelet drugs	No	284 (84.02)	288 (85.21)	1.18	8.222	0.042*
	Poor	11 (3.25)	12 (3.55)	0.30		
	Moderate	27 (7.99)	12 (3.55)	−4.44		
	Good	16 (4.73)	26 (7.69)	2.96		

*P < 0.05.

### 3.5 Comparison of activation among high-risk stroke patients before and after intervention

**FIGURE 3:**
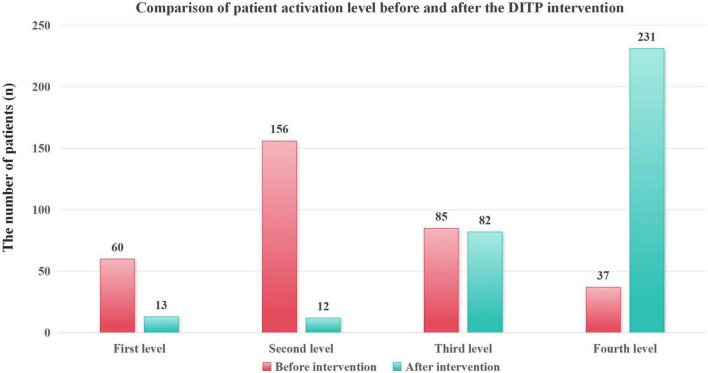
Comparison of patient activation levels before and after the DITP intervention.

After 6 months of intervention, the patient’s activation score increased from 54.97 ± 9.08 points to 75.93 ± 15.99 points (*t* = −21.016, *P* < 0.001), and the level of patient activation improved significantly after intervention (χ^2^ = 294.176, P < 0.05), as shown in Table 7 and Figure 3.

**TABLE 7:** Comparison of patient activation before and after intervention.

Characteristic	Category	Before intervention	After intervention	t/χ^2^	*P*
Patient activation score ($\bar{x}$ s)		54.97 ± 9.08	75.93 ± 15.99	t = −21.016	< 0.001*
Patient activation level N (%)	First level	60 (17.75)	13 (3.85)	χ^2^ = 294.176	< 0.001*
	Second level	156 (46.15)	12 (3.55)		
	Third level	85 (25.15)	82 (24.26)		
	Fourth level	37 (10.95)	231 (68.34)		

*P < 0.05.

### 3.6 Incidence of major adverse cardiovascular events in high-risk stroke patients after intervention

As shown in Table 8, the incidence of myocardial infarction in the high-risk stroke population was 0.59% (2/338 people), the incidence of cerebrovascular ischemic events was 3.85% (13/338 people), and no other adverse events occurred after 6 months of intervention.

**TABLE 8:** Incidence of major adverse cardiovascular events in the high-risk stroke population.

Characteristic	Frequency	Percent (%)
Malignant arrhythmia	0	0.00
Myocardial infarction	2	0.59
Sudden cardiac death	0	0.00
Revascularization	0	0.00
Cerebrovascular ischemic events	13	3.85

### 3.7 Single-factor analysis of the optimal control rate of risk factors

As shown in Table 9, the factors influencing patients’ ability to achieve the optimal control criteria for stroke risk factors were analyzed. The results of univariate analysis revealed that employment status (χ^2^ = 5.201, *P* = 0.023), per capita monthly income of family (χ^2^ = 6.386, *P* = 0.041), hypertension status (χ^2^ = 4.774, *P* = 0.029), diabetes status (χ^2^ = 16.535, *P < 0.001*), family history of stroke (χ^2^ = 5.74, *P* = 0.017), hypoglycemic treatment (χ^2^ = 6.769, *P* = 0.029) and lipid lowering treatment (χ^2^ = 9.473, *P* = 0.002) significantly affected the optimal control of risk factors.

**TABLE 9:** Single-factor analysis of optimal control of risk factors.

Characteristic	Category	Optimal controlled (n)	Not optimal controlled (n)	χ^2^	*P*-value
Sex	Male	10	139	1.543	0.214
	Female	20	169		
Long term residence	Rural area	1	6	0.259	0.611
	City	29	302		
Age (years)	65–74	27	245	1.901	0.168
	75–79	3	63		
Marital status	Married	29	290	0.024	0.877
	Widowed/divorced/Single	1	18		
Education degree	Junior high school and below	25	242	0.374	0.541
	High school and above	5	66		
Employment status	Currently working	10	51	5.201	0.023*
	Retired	20	257		
Per capita monthly household income (yuan)	≤ 4,000	26	261	6.386	0.041*
	> 4,000	4	47	0.000	0.989
Medical insurance type	Urban resident medical insurance	22	245	2.508	0.285
	Urban employee medical insurance	3	38		
	New rural cooperative medical insurance/Others	5	25		
Drink alcohol	yes	3	55	1.187	0.276
	no	27	253		
Overweight/obesity	yes	22	223	0.012	0.913
	no	8	85		
Moderate physical activity	yes	14	100	2.466	0.116
	no	16	208		
Hypertension	yes	22	270	4.774	0.029*
	no	8	38		
Diabetes	yes	5	171	16.535	< 0.001*
	no	25	137		
Dyslipidemia	yes	22	246	0.711	0.399
	no	8	62		
Atrial fibrillation/valvular heart disease	yes	1	7	0.133	0.715
	no	29	301		
Family history of stroke	yes	12	64	5.74	0.017*
	no	18	244		
antihypertensive therapy	yes	18	194	0.119	0.730
	no	12	114		
Hypoglycemic therapy	yes	5	126	6.769	0.009*
	no	25	182		
Lipid-lowering therapy	yes	16	82	9.473	0.002*
	no	14	226		
Antiplatelet therapy	yes	6	37	1.571	0.210
	no	24	271		
Medication adherence level of antihypertensive drugs	Not taking medication	12	105	1.001	0.801
	Poor medication adherence	4	32		
	Moderate medication adherence	7	94		
	Good medication adherence	7	77		
Medication adherence level of hypoglycemic drugs	Not taking medication	16	221	5.447	0.142
	Poor medication adherence	3	20		
	Moderate medication adherence	5	22		
	Good medication adherence	6	45		
Medication adherence level of hypolipidemic drugs	Not taking medication	16	221	5.447	0.142
	Poor medication adherence	3	20		
	Moderate medication adherence	5	22		
	Good medication adherence	6	45		
Medication adherence level of antiplatelet drugs	Not taking medication	23	265	5.798	0.122
	Poor medication adherence	0	12		
	Moderate medication adherence	2	10		
	Good medication adherence	5	21		
Patient activation level	First level	0	13	7.73	0.052
	Second level	1	11		
	Third level	2	80		
	Fourth level	27	204		

*P < 0.05.

### 3.8 Multivariate logistic regression analysis of the optimal control rate of risk factors

There was no multicollinearity among the influencing factors (tolerance > 0.1 and VIF < 15), as shown in Table 10. Multivariate logistic regression analysis revealed that the optimal control rate for stroke risk factors in retired patients was 0.264 times higher than that in unemployed patients (95% CI = 0.094–0.745, *P* = 0.012). The optimal control rate of risk factors for high-risk stroke patients with diabetes was 0.079 times greater than that for those without diabetes (95% CI = 0.013–0.481, *P* = 0.006). The optimal control rate for risk factors in patients with a family history of stroke was 2.736 times higher than that in those without a family history (95% CI = 1.050–7.131, *P* = 0.039). The optimal control rate for risk factors in patients receiving lipid-lowering therapy was 9.176 times greater than that in those not receiving such treatment (95% CI = 3.178–26.496, *P* < 0.001). The specific results are shown in Table 11.

**TABLE 10:** Multiple linear regression analysis.

Characteristic	Tolerance	VIF
Sex	0.964	1.038
Age (years)	0.926	1.080
Long term residence	0.922	1.085
Marital status	0.928	1.077
Medical insurance type	0.907	1.103
Education degree	0.941	1.062
Employment status	0.970	1.031
Per capita monthly household Income (yuan)	0.921	1.086
Hypertension	0.958	1.044
Diabetes	0.470	2.127
Dyslipidemia	0.920	1.087
Family history of stroke	0.927	1.078
Hypoglycemic therapy	0.476	2.102
Lipid lowering therapy	0.912	1.096

**TABLE 11:** Multivariate logistic regression analysis for optimal control of risk factors.

Characteristic	Classification	OR	95% CI	*P*-value
**Sex**	Female			
	Male	0.545	0.213–1.395	0.205
**Age (years)**	65–74			
	75–79	0.385	0.094–1.587	0.187
**Long term residence**	Rural area			
	City	1.711	0.074–39.655	0.738
**Marital status**	Widowed/divorced/single			
	Married	1.937	0.178–21.051	0.587
**Medical insurance type**	Urban employee medical insurance			
	Urban resident medical insurance	1.632	0.367–7.252	0.520
	New rural cooperative medical insurance/Others	3.742	0.874–16.030	0.075
**Education degree**	Junior high school and below			
	High school and above	0.718	0.212–2.435	0.718
**Employment status**	Currently working			
	Retired	0.264	0.094–0.745	0.012*
**Per capita monthly household income (yuan)**	≤ 4,000			
	> 4,000	1.719	0.449–6.582	0.429
**Hypertension**	No			
	Yes	0.459	0.155–1.354	0.158
**Diabetes**	No			
	Yes	0.079	0.013–0.481	0.006*
**Dyslipidemia**	No			
	Yes	0.334	0.111–1.004	0.051
**Family history of stroke**	No			
	Yes	2.736	1.050–7.131	0.039*
**Hypoglycemic therapy**	No			
	Yes	1.407	0.211–9.396	0.724
**Lipid lowering therapy**	No			
	Yes	9.176	3.178–26.496	< 0.001*

*P < 0.05.

## 4 Discussion

This study included a total of 338 high-risk stroke patients from community health service centers in Liaoning Province, China, and we conducted a 6-month intervention using the DITP model in primary healthcare. This model involves a patient-centered, team-based approach and a digital platform to develop individualized treatment plans and follow-up schedules for patients at high risk of stroke. After professional training, primary care general practitioners were proficient in primary care prevention and treatment techniques for multiple risk factors for stroke and were better able to provide patients with comprehensive and continuous health services.

For the present study, we evaluated the application effect of this model by statistically analyzing the control compliance of multiple risk factors for stroke before and after intervention, changes in physiological indicators, medication adherence, patient activation, and the incidence of major adverse cardiovascular events after intervention. The prevalence rates of chronic diseases were as follows: hypertension (89.94%), dyslipidemia (73.37%), overweight (65.70%), diabetes (52.96%), and atrial fibrillation or valvular heart disease (2.07%). Among them, 86.7% of patients had two or more chronic diseases at the same time, and 27.22% had a history of smoking. After 6 months of intervention, the blood pressure and blood lipid compliance rates improved with respect to the control of multiple risk factors, whereas the blood glucose compliance rate (50.30% preintervention vs. 62.43% postintervention) and the optimal risk factor control rate (3.85% preintervention vs. 8.88% postintervention) significantly improved. The levels of the physiological indicators FBG and TC significantly decreased, whereas the levels of TG and LDL-C decreased slightly. Patient medication adherence and patient motivation obviously improved. During follow-up, myocardial infarction occurred in 2 patients (0.59%), and cerebral ischemic events occurred in 13 patients (3.85%). The analysis revealed that employment status, family per capita monthly income, hypertension status, diabetes history, family history of stroke, and acceptance of hypoglycemic and lipid-lowering treatment significantly affected the optimal control rate of risk factors. Specifically, retired individuals, patients without diabetes, those with a family history of stroke, and those receiving lipid-lowering treatment had better optimal control of risk factors.

With the aging of the population, the prevalence of diabetes, hypertension and hyperlipidemia is increasing, and there is a problem of poor control; the health burden of stroke patients in China is also increasing (22). In this study, the preintervention prevalence rates of hypertension [89.94% in this study vs. 27.5% in Zhang M and other studies (23)], diabetes [52.96% in this study vs. 11.7% in Jin C and other studies (24)] and dyslipidemia [73.37% in this study vs. 8.9% in Hu Y and other studies (25)] among patients at the primary care level in Liaoning Province were higher than those reported in Chinese adults in previous studies. This may be related to the heavy oil and high salt diet in Northeast China and the cold climate environment, leading to a higher prevalence of hypertension, diabetes and dyslipidemia in this study population. After 6 months of intervention via the DITP at the primary care level, the control compliance rates for hypertension (46.45% in this study vs. 37.0% in Sun L and other research results (26)), dyslipidemia [18.93% in this study vs. 5.4% Xia Q and other research results (27)], and diabetes FBG [62.43% in this study vs. Deng Q and other research results 34.6% (28)] in the high-risk population for stroke improved compared with those before the intervention and were superior to those reported in other domestic studies. The above improvement in the compliance rate reflects the superiority of the people-centered concept in this intervention model, which focuses on the comprehensive needs of patients, including understanding their thoughts, concerns, and expectations and paying attention to the impact of health issues on their lives. At the same time, based on the assessment of the patient’s social background, general practitioners communicate fully with the patient, make joint decisions between doctors and patients, and develop individualized treatment plans for the patient, improving compliance with medical treatment.

Although the optimal control rate for risk factors in this study increased from the initial 3.85 to 8.88% after intervention, it was still far lower than the improvement rate of 36.2% after 6 months of intervention in the multicenter CREST-1 study in the United States (20). Therefore, the control level of stroke risk factors in China still needs to be further improved, and primary care prevention and treatment are crucial. At present, regarding the primary management of stroke prevention, while improving the compliance rate of individual risk factors for stroke, we should also pay more attention to the implementation and promotion of a comprehensive control model for multiple risk factors at the primary care level (13–15).

Drug compliance is closely related to the geographical environment, economic level, patient education level, and lifestyle of the study population (29). The proportion of patients with good adherence to antihypertensive medication after intervention in this study was 24.85%, which is similar to the proportion of 22.3% reported by Shiraly et al. (30). The compliance score for patients taking hypoglycemic drugs was 7.19 ± 1.24 points, which was significantly higher than the 5.43 ± 1.8 points reported by Gao et al. in China (31). This reflects the greater level of importance attached to hypertension and diabetes among the primary care patients in this study. Although there has been an improvement in medication adherence to lipid-lowering drugs after intervention, the proportion of good medication adherence remains low at 21.89%, which was 69.0% in Farsaei et al.’s study (32); this is due to insufficient awareness of blood lipid abnormalities among primary care patients, who are more likely to self-discontinue medication because they are asymptomatic and have decreased blood lipid levels. In addition, some patients refuse to take lipid-lowering drugs because of concerns about their adverse effects on the body. Moreover, the proportion of patients with good adherence to antiplatelet medication after intervention was 7.69%, which was also much lower than the 64.9% reported in Akao et al.’s study (33); this may be related to patients’ concerns about bleeding risk and self-discontinuation of medication due to perceived asymptomatic status. In the future, general practitioners should strengthen their management of patients’ medication compliance.

Patient motivation is closely related to good health-related behaviors (exercise and a healthy diet), quality of life, and health satisfaction (34). After the intervention in this study, the patient’s activation score and level significantly improved, with the proportion of high-level activation increasing from 10.95 to 68.34%. The postintervention cumulative score was 75.93 ± 15.99 points, which was higher than the activation score of 61.9 ± 18.0 points obtained by van Meijeren Pont et al. after 6 months of intervention in stroke patients (35). The combination of online and offline management models in this study has made communication between general practitioners and patients more convenient and increased patients’ trust in doctors. Through the joint efforts of the comprehensive hospital and the team of primary care general practitioners, chronic disease-related knowledge is explained, and health education is provided to patients, allowing them to promptly and thoroughly be awake of their own disease conditions while developing personalized diet plans, exercise plans, and health management plans. Patients should be encouraged to develop healthy behaviors, seek medical advice reasonably, and follow the doctor’s health advice and disease diagnosis and treatment plan; this enhances patients’ understanding of and confidence in controlling stroke risk factors and improves their motivation.

Moreover, this study revealed that the best control of risk factors was better in patients who were currently working, did not suffer from diabetes, had a family history of stroke, and were treated with lipid-lowering therapy; this confirms the findings of de Havenon et al. (36) that unemployment is one of the social determinants of increased stroke mortality in the elderly population (36). This finding also verifies the findings of Błaż and Sarzyńska-Długosz (37) that a family history of stroke is one of the risk factors for increasing the risk of stroke in the primary prevention of stroke (37). In China, the prevalence of diabetes is as high as 11.7% (24), and the prevalence of dyslipidemia is as high as 35.6% (21). Effective comprehensive interventions for high-risk stroke patients with diabetes and dyslipidemia can reduce their stroke risk (13–15). At present, stroke has become a public health issue, and the task of stroke prevention and treatment at the primary care level in China is arduous and still faces enormous challenges. We need to strengthen health education for high-risk populations for stroke while also paying attention to evaluating the social determinants of patients. To better address this health challenge, public awareness of the primary prevention of stroke should be strengthened, primary healthcare services should be optimized, and the treatment and compliance rates for stroke risk factors should be improved.

## 5 Strengths and limitations

The advantage of this study is that it first focuses on the comprehensive control of stroke risk factors and their impact on the primary prevention of stroke. A hospital digital information platform has been constructed with intelligent technology, and the DITP model has been developed. For the first time, in China, Ariadne principles and methods have been applied to intervene in multiple risk factors for stroke, thereby improving the control of risk factors, detecting the occurrence of cardiovascular and cerebrovascular adverse events in a timely manner, and enhancing the effectiveness of health management for high-risk stroke patients.

This study also has limitations. First, the intervention period of this study was 6 months, and the intervention process may also be influenced by climate and geography. Liaoning Province is located in the northeastern region of China, and in the later stage of the intervention, it entered the cold winter season, which led to a decrease in outdoor activities for residents. Therefore, in this study, the appropriate physical activity rate and BMI qualification rate decreased compared with those before the intervention. We will continue to conduct intervention research in the future to better evaluate the benefits of this management model for high-risk elderly stroke patients. During the research period, there may also be changes in national guidelines or other healthcare utilization factors, which will be accounted for in subsequent studies.

## 6 Conclusion

The DITP model effectively improved the stroke risk factor control rate, physiological indicators, medication compliance, and patient activation in the elderly high-risk population for stroke and detected the occurrence of major adverse cardiovascular events in a timely manner. This model will help actively improve the health of elderly high-risk stroke patients in the long term and should be further applied and promoted in primary health care in the future.

## Data Availability

The raw data supporting the conclusions of this article will be made available by the authors, without undue reservation.
